# Upper GI Endoscopy in Resource‐Constrained Settings: Bridging the Gap

**DOI:** 10.1002/jgh3.70089

**Published:** 2025-01-03

**Authors:** Muhammad Uwais Ashraf, Madhumita Premkumar

**Affiliations:** ^1^ Department of Hepatology Postgraduate Institute of Medical Education and Research Chandigarh India

**Keywords:** endoscopy, gastroduodenoscopy, resource‐constrained

**Abbreviations**
ERCP: endoscopic retrograde cholangiopancreatography
ES: endoscopic ultrasound
GI: gastrointestinal
GIE: GI endoscopy

Upper gastrointestinal (GI) endoscopy is an essential adjunct to gastroenterology and hepatology practice, providing invaluable diagnostic and therapeutic capabilities for patients with GI diseases [1]. Therapeutic gastrointestinal endoscopy (GIE) is required for the management of acute GI bleeding, including portal hypertension or ulcer‐related bleeding. It is also needed in patients who require post‐pyloric feeding, GI malignancy, and for management of benign and malignant strictures. Colonoscopy is needed for screening of large bowel cancers, inflammatory bowel disease, and GI tuberculosis. However, in resource‐limited settings, this essential procedure remains out of reach for millions of patients [2].

As global health inequities persist, we must address the challenges and explore innovative solutions to bring basic GIE service‐gastroduodenoscopy and colonoscopy to those who need it the most. Although advanced endoscopic procedures like endoscopic retrograde cholangiopancreatography and endoscopic ultrasound remain in the specialist domain, basic endoscopy should be accessible at sites where surgical training is provided, with opportunities for training fellows in medicine and surgical units in workshops to provide GIE services in West Africa [3, 4, 5].

In this issue of JGH Open, Nziku et al. describe their experience of providing endoscopy services in Tanzania [6]. In Tanzania, 4.3 gastroenterologists are practicing per 10 000 000 people [7]. The delivery of GIE procedures, both diagnostic and therapeutic, and patient outcomes are not well described in the literature. Rebleeding in this study occurred in 40.1% of patients as rebleeding was higher in patients who received conservative treatment (72.0%) compared with endoscopic treatment.

In another large study from Zanzibar on 3146 patients, gastro‐duodenitis, peptic ulcer disease are the most common endoscopic diagnoses in Zanzibar [8]. The presence of *H. pylori* was significantly associated with duodenal ulcer and gastric cancer. In another study conducted by Pan‐African Academy of Christian Surgeons sites in rural Africa, 20 surgical trainees performed a total of 2181 GIE procedures [3]. Of all procedures, 546 (26.7%) involved cancer or mass, 267 (12.2%) involved a report of blood loss, and 452 (20.7%) reported pain as a component of the diagnosis. Interventions beyond biopsy were reported in 555 (25%) procedures. This reflects the need for better delivery of GIE services in a rural setting, with the establishment of better training facilities.

In a study from Nigeria, rebleeding rates were low following endoscopic therapy (5.5%) and were expectedly higher in patients who had conservative treatment (75.0%) [9]. As such the present study attempts to bridge the gap in the requirement of specialist GI care in a resource‐constrained setting and provides useful insight for health policy.

The cost‐effectiveness of implementing upper GI endoscopy in resource‐limited settings cannot be overstated. Conditions like upper GI bleeding, both variceal as well as non‐variceal, can be life‐threatening. Early detection of gastric cancer, a leading cause of cancer‐related deaths in many low‐ and middle‐income countries, can significantly improve patient outcomes and reduce the overall burden on healthcare systems [10]. Moreover, the ability to diagnose and treat conditions such as peptic ulcer disease and variceal bleeding can prevent costly complications and save lives [11].

In resource‐constrained environments, there is limited access to expensive endoscopic equipment [12], inadequate infrastructure, and a shortage of trained personnel are just a few of the hurdles that must be overcome. Moreover, the lack of maintenance support and reliable supply chains for consumables further complicates the sustainable implementation of endoscopy services [13].

Innovative approaches are emerging that could revolutionize the delivery of upper GI endoscopy in low‐resource settings. Portable, battery‐operated endoscopes are being developed, offering a more affordable and practical alternative to traditional tower‐based systems [14]. Like a standard nasogastric (NG) tube, this endoscope system features a slim probe that can be easily inserted through the nasal passage to reach the stomach for diagnostic purposes. The probe tip, matching the width of a 16‐French NG tube, connects to an even slimmer 3.6 mm shaft. Thanks to its NG tube‐like design, healthcare providers can operate this system without specialized endoscopic training. Telemedicine platforms enable remote guidance and interpretation, extending the reach of specialist expertise. In addition, novel disinfection methods are being explored to address the critical issue of equipment sterilization in areas with unreliable water and electricity supplies [15]. However, there remains a need for training and familiarity for general practitioners and a supported network for referral of cases for diagnostic and therapeutic endoscopy. In particular, the use of GI simulators to familiarize trainees with the endoscopy equipment is useful for training residents and encourage them to acquire skills to perform these vital procedures [16, 17].

Training and capacity building must be at the forefront of any effort to expand endoscopy services. Partnerships between academic institutions in high‐ and low‐income countries can facilitate knowledge transfer and skill development. Short‐term training programs, coupled with ongoing mentorship, can create a sustainable workforce capable of performing and interpreting upper GI endoscopies safely and effectively [18].

In conclusion, while the challenges of implementing upper GI endoscopy in resource‐limited settings are significant, they are not insurmountable. Through innovation, education, and collaboration, we can bridge the diagnostic gap and ensure that this life‐saving procedure is available to all, regardless of geographical or economic constraints.

## Conflicts of Interest

Dr. Madhumita Premkumar is a member of the editorial board of JGHOpen and was excluded from all decisions related to this article during the entire editorial process.
